# Racial disparities in cancer-related survival in patients with squamous cell carcinoma of the esophagus in the US between 1973 and 2013

**DOI:** 10.1371/journal.pone.0183782

**Published:** 2017-08-23

**Authors:** Alice Kim, Peter Ashman, Melissa Ward-Peterson, Juan Manuel Lozano, Noël C. Barengo

**Affiliations:** 1 Herbert Wertheim College of Medicine, Florida International University, Miami, Florida, United States of America; 2 Department of Medical and Health Science Research, Herbert Wertheim College of Medicine, Florida International University, Miami, Florida, United States of America; 3 Department of Epidemiology, Robert Stempel College of Public Health & Social Work, Florida International University, Miami, Florida, United States of America; Baylor College of Medicine, UNITED STATES

## Abstract

**Background:**

Esophageal cancer makes up approximately 1% of all diagnosed cancers in the US. There is a persistent disparity in incidence and cancer-related mortality rates among different races for esophageal squamous cell carcinoma (SCC). Most previous studies investigated racial disparities between black and white patients, occasionally examining disparities for Hispanic patients. Studies including Asians/Pacific Islanders (API) as a subgroup are rare. Our objective was to determine whether there is an association between race and cancer-related survival in patients with esophageal SCC.

**Methods and findings:**

This was a retrospective cohort study using the National Cancer Institute’s Surveillance, Epidemiology, and End Result (SEER) database. The SEER registry is a national database that collects information on all incident cancer cases in 13 states of the United States and covers nearly 26% of the US population Patients aged 18 and over of White, Black, or Asian/Pacific Islander (API) race with diagnosed esophageal SCC from 1973 to 2013 were included (n = 13,857). To examine overall survival, Kaplan-Meier curves were estimated for each race and the log-rank test was used to compare survival distributions. Cox proportional hazards models were used to estimate unadjusted and adjusted hazard ratios with 95% confidence intervals. The final adjusted model controlled for sex, marital status, age at diagnosis, decade of diagnosis, ethnicity, stage at diagnosis, and form of treatment. Additional analyses stratified by decade of diagnosis were conducted to explore possible changes in survival disparities over time. After adjustment for potential confounders, black patients had a statistically significantly higher hazard ratio compared to white patients (HR 1.08; 95% confidence interval (CI) 1.03–1.13). However, API patients did not show a statistically significant difference in survival compared with white patients (HR 1.00; 95% CI 0.93–1.07). Patients diagnosed between 1973 and 1979 had twice the hazard of death compared to those diagnosed between 2000 and 2013 (HR 2.05, 95% CI 1.93–2.19). Patients diagnosed in 1980–1989 and 1990–1999 had had HRs of 1.59 (95% CI 1.51–1.68) and 1.33 (95% CI 1.26–1.41), respectively. After stratification according to decade of diagnosis, the HR for black patients compared with white patients was 1.14 (95% CI 1.02–1.29) in 1973–1979 and 1.12 (95% CI 1.03–1.23) in 1980–1989. These disparities were not observed after 1990; the HR for black patients compared with white patients was 1.03 (95% CI 0.93–1.13) in 1990–1999 and 1.05 (95% CI 0.96–1.15) in 2000–2013.

**Conclusions:**

Black patients with esophageal SCC were found to have a higher hazard of death compared to white and API patients. Survival disparities between races appear to have decreased over time. Future research that takes insurance status and other social determinants of health into account should be conducted to further explore possible disparities by race.

## Introduction

Racial and ethnic minorities have been shown to have worse outcomes and receive a lower quality of healthcare than those of non-minorities. This has been attributed to decreased access to necessary health care due to socioeconomic status, language, geography, and cultural familiarity. There is a great need to educate healthcare professionals regarding racial disparities in order to better provide quality healthcare to minority patients [1].

Esophageal cancer makes up approximately 1% of all diagnosed cancers in the US [2]. Much of the advances in treatment options involving multimodality therapy—such as radiation, chemotherapy, or both before surgery—was developed in the mid-1980s. Despite improved treatment and survival rates, however, the 5-year relative survival has remained at 19% since 2000 [3–5].

Although there are declining overall mortality rates in cancer patients in the US, there is a persistent disparity in incidence and cancer-related mortality rates among different races/ethnicities for esophageal squamous cell carcinoma (SCC). In the US, black patients have more than fivefold higher incidence (16.8 per 100,000) of esophageal SCC than white patients (3 per 100,000) [6]. Black patients were more likely to be diagnosed at an advanced stage and less likely to have surgery compared to white patients [7, 8]. Studies describing esophageal cancer epidemiology found that black patients have a higher cancer-related mortality than white patients [9, 10, 11].

Most previous retrospective cohort studies investigated racial disparities between black and white patients [7, 9, 10–15], occasionally examining disparities for Hispanic patients [11]. Studies including Asians/Pacific Islanders (API) as a subgroup are rare [16, 17]. One of the few studies of esophageal cancer among API patients was done from 1930–1967, showing that Chinese and Japanese males had a higher all-cause mortality compared to white patients [16].

The objective of this study was to determine whether there are racial disparities in cause-specific survival among patients with esophageal SCC, and whether such disparities have changed over the last four decades.

## Materials and methods

### Study design

This was a retrospective cohort study and secondary data analysis using the National Cancer Institute’s Surveillance, Epidemiology, and End Result (SEER) database (S1 File). SEER registry is a national database that collects information on all incident cancer cases in 13 states of the United States and covers nearly 26% of the US population [18]. Adults ages 18 and over in 13 states diagnosed with SCC of the esophagus (ICD-O-3) from 1973 to 2013 with information on stage at diagnosis were included. After excluding those patients with a race category of “Other”, an unknown stage at diagnosis, and those without a primary tumor of esophageal SCC of the esophagus we were left with a sample size of 13,857. The main exposure of interest was race, defined as white, black, or API. The main outcome was survival using cause-specific mortality. Possible confounders included sex, age at diagnosis (18–49, 50–59, 60–69, 70–79, and 80 years or older), decade of diagnosis (1973–1979, 1980–1989, 1990–1999, and 2000–2013), ethnicity (non-Spanish/Hispanic/Latino and Spanish/Hispanic/Latino), stage at diagnosis (in-situ, localized, regional, or distant), form of treatment (radiation and surgery), marital status, and insurance status. Marital status was categorized as partnered (married or domestic partner) and un-partnered (single, separated, divorced, or widowed).

### Statistical analysis

**Fig 1:**
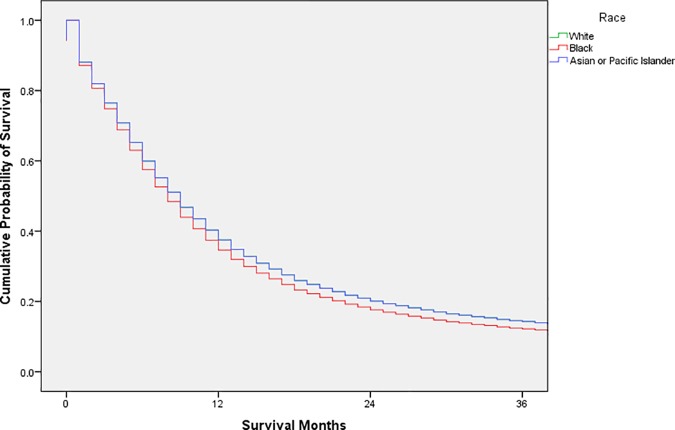
Adjusted survival curves, by race, for adult patients in the SEER database diagnosed with esophageal squamous cell carcinoma, 1973–2013.

**Fig 2:**
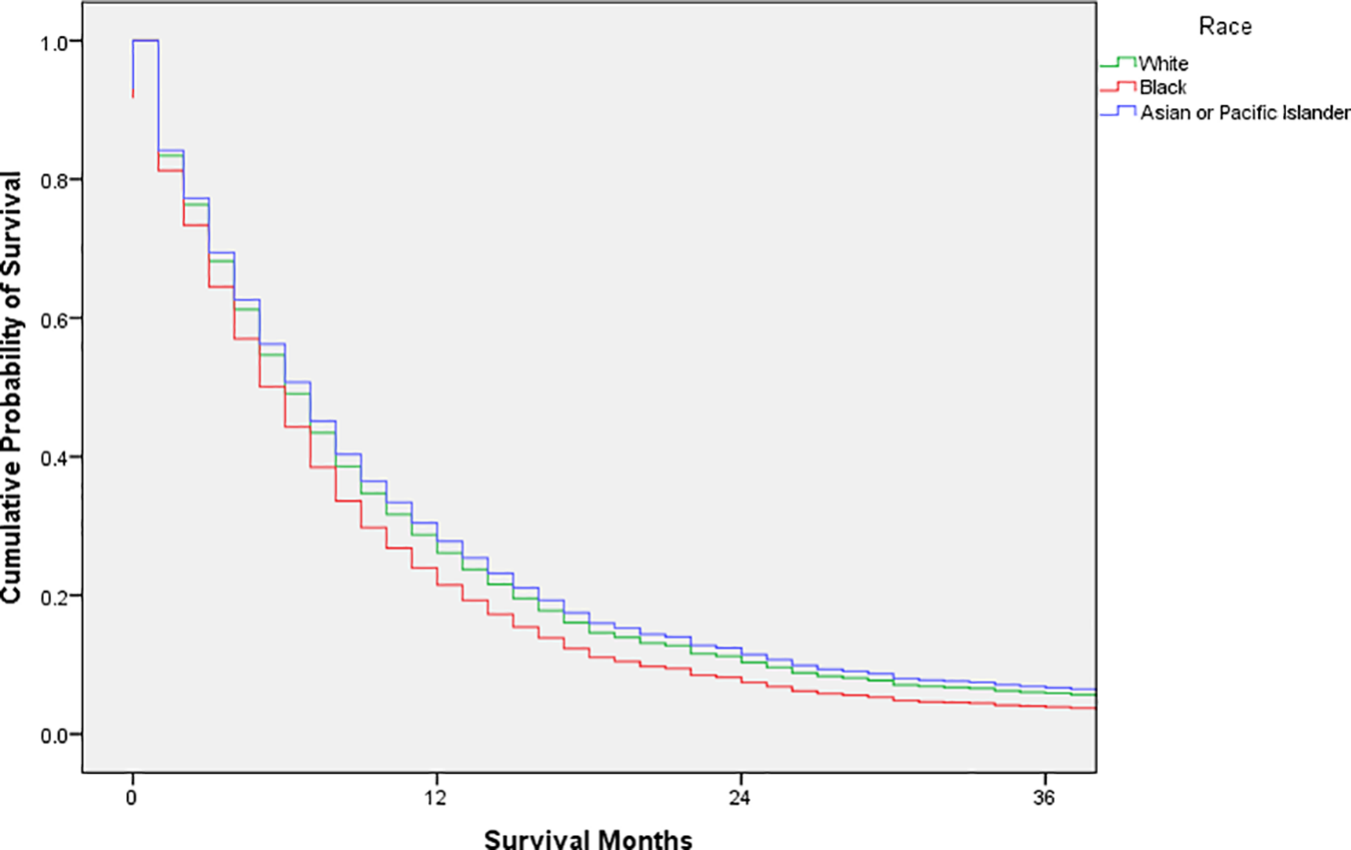
Adjusted survival curves, by race, for adult patients in the SEER database diagnosed with esophageal squamous cell carcinoma, 1970–1979.

**Fig 3:**
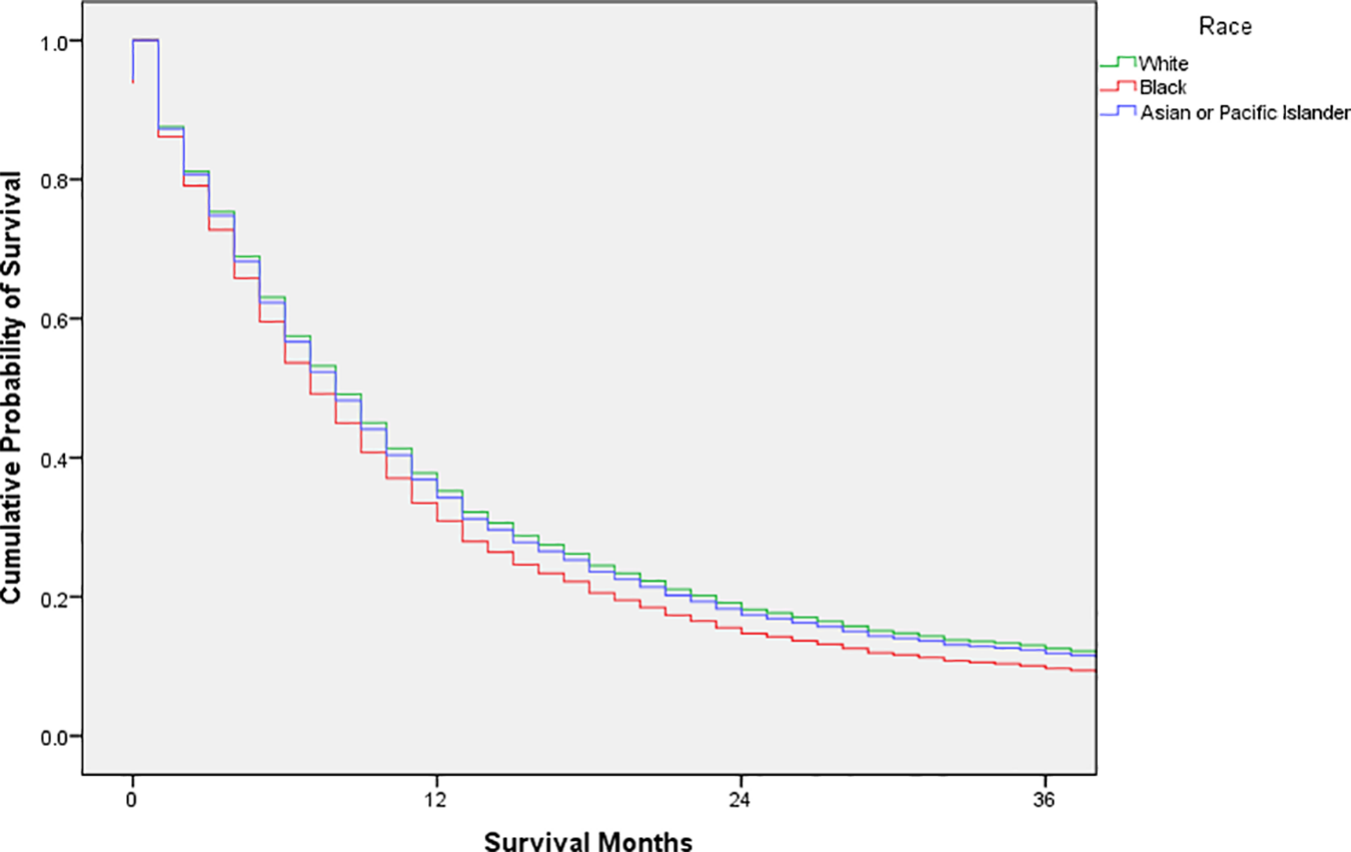
Adjusted survival curves, by race, for adult patients in the SEER database diagnosed with esophageal squamous cell carcinoma, 1980–1989.

**Fig 4:**
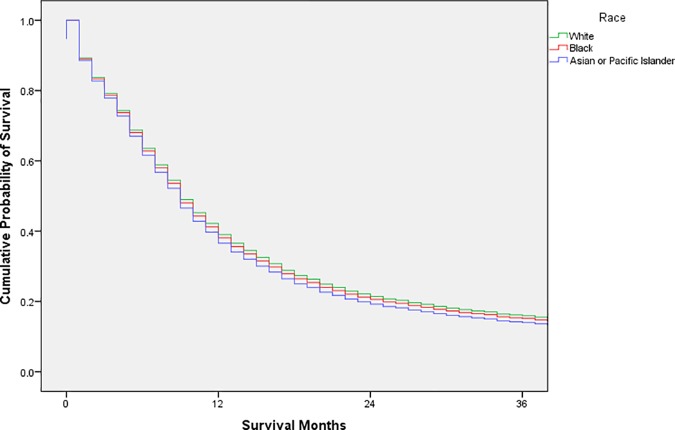
Adjusted survival curves, by race, for adult patients in the SEER database diagnosed with esophageal squamous cell carcinoma, 1990–1999.

**Fig 5:**
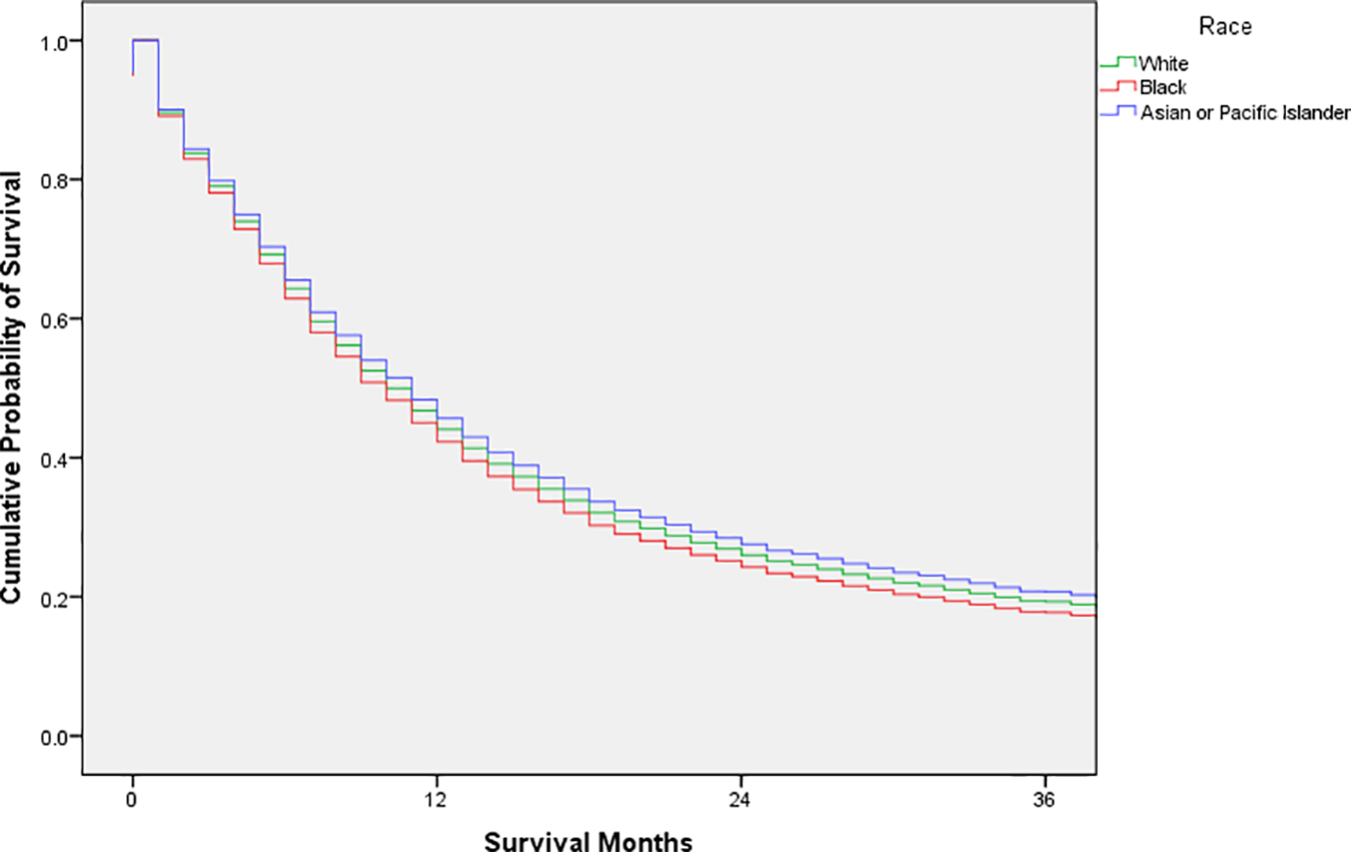
Adjusted survival curves, by race, for adult patients in the SEER database diagnosed with esophageal squamous cell carcinoma, 2000–2013.

Exploratory analysis was conducted using frequency distributions. Chi-square tests were used to compare the distribution of possible confounders by race. Cox proportional hazards models were used to estimate unadjusted and adjusted hazard ratios with 95% confidence intervals; adjusted survival curves were generated from the adjusted analysis (Figs 1, 2, 3, 4 and 5). Variance inflation factors were used to assess for multicollinearity in the final adjusted model; the proportional hazards assumption was tested graphically. The final adjusted model controlled for sex, marital status, age at diagnosis, decade of diagnosis, ethnicity, stage at diagnosis, and form of treatment. Additional analyses stratified by decade of diagnosis were conducted to explore possible changes in survival disparities over time. SPSS 23 (IBM, Armonk, New York) was used for all analyses. P-values less than or equal to 0.05 were considered statistically significant.

### Ethical considerations

Permission to use and access to the SEER database was obtained through the SEER website. Ethical approval was waived since the analysis was considered nonhuman subjects research by the Florida International University Health Sciences IRB.

## Results

Table 1 describes the demographic and clinical characteristics patients with esophageal SCC in the SEER registry from 1973 to 2013. Among all races, most patients were diagnosed between the ages of 60 and 69 years old (white 34.2%, black 33.5%, API 32.5%). However, a greater proportion of younger black patients (18–49 years-old) were diagnosed with esophageal SCC compared to other races. A higher proportion of men were diagnosed with esophageal SCC; API women represented the lowest proportion of cases (18.6%). Increasing proportions of cases among API patients were observed over time. In contrast to white and API patients, a higher proportion of black patients were un-partnered (65.5%), uninsured (7.0%), receiving Medicaid (32.6%), and diagnosed at a later stage (36.4%). More than two-thirds of patients across all races received radiation, while less than a third underwent surgery.

**Table 1:** Demographic and clinical characteristics, by race, of adult patients in the SEER registry diagnosed with esophageal squamous cell carcinoma, 1973–2003.

	Race			
	White	Black	Asian/Pacific Islander	p-value
	N (%)	N (%)	N (%)	
**Age (years)**				<0.001
18–49	406 (4.7)	544 (13.7)	94 (7.4)	
50–59	1653 (19.0)	1317 (33.2)	257 (20.2)	
60–69	2970 (34.2)	1328 (33.5)	415 (32.5)	
70–79	2524 (29.1)	635 (16.0)	335 (26.3)	
> = 80	1132 (13.0)	139 (3.5)	174 (13.6)	
**Sex**				<0.001
Male	5638 (64.9)	2856 (72.1)	1038 (81.4)	
Female	3047 (35.1)	1107 (27.9)	237 (18.6)	
**Decade of diagnosis**				<0.001
1973–1979	1696 (19.5)	670 (16.9)	145 (11.4)	
1980–1989	2331 (26.8)	1219 (30.8)	247 (19.4)	
1990–1999	2076 (23.9)	1031 (26.0)	308 (24.2)	
2000–2013	2582 (29.7)	1043 (26.3)	575 (45.1)	
**Marital Status**				<0.001
Unpartnered^a^	3623 (43.1)	2436 (65.5)	436 (35.1)	
Partnered^b^	4788 (56.9)	1283 (34.5)	806 (64.9)	
**Ethnicity**				<0.001
Non-Spanish/Hispanic/Latino	8209 (94.5)	3947 (99.6)	1262 (99.0)	
Spanish/Hispanic/Latino	476 (5.5)	16 (0.4)	13 (1.0)	
**Stage at Diagnosis**				<0.001
In-situ	93 (1.1)	16 (0.4)	10 (0.8)	
Localized	2984 (34.4)	1250 (31.5)	319 (25.0)	
Regional	2950 (34.0)	1253 (31.6)	488 (38.3)	
Distant	2658 (30.6)	1444 (36.4)	458 (35.9)	
**Received Radiation**				0.314
Yes	5734 (67.4)	2556 (66.5)	870 (68.7)	
No	2775 (32.6)	1290 (33.5)	397 (31.3)	
**Received Surgery**				<0.001
Yes	2378 (30.1)	982 (26.0)	346 (27.5)	
No	5525 (69.9)	2799 (74.0)	912 (72.5)	

^a^Unpartnered: single, separated, divorced or widowed;

^b^Partnered: married or unmarried/domestic partner

The adjusted cumulative survival curve of the adjusted Cox regression model revealed a lower survival for esophageal SCC in black patients (Fig 1).

Table 2 shows unadjusted and adjusted hazard ratio (HR) estimations. After adjustment for potential confounders, black patients had a statistically significantly higher hazard ratio compared to white patients (HR 1.08; 95% confidence interval (CI) 1.03–1.13). However, API patients did not show a statistically significant difference in survival compared with white patients (HR 1.00; 95% CI 0.93–1.07). Additionally, as age at diagnosis increased, hazard ratios increased. Females had a lower hazard of death compared with males (HR 0.87; 95% CI 0.83–0.91). Un-partnered patients had an increased hazard compared with those who were partnered (HR 1.17; 95% CI 1.12–1.22). As the stage of diagnosis increased, the hazard of death increased. Patients who did not receive radiation therapy or undergo surgery had HRs of 1.78 (95% CI 1.70–1.87) and 2.17 (95% CI 2.05–2.28), respectively. Patients diagnosed between 1973 and 1979 had twice the hazard of death compared to those diagnosed between 2000 and 2013 (HR 2.05, 95% CI 1.93–2.19). Patients diagnosed in 1980–1989 and 1990–1999 had had HRs of 1.59 (95% CI 1.51–1.68) and 1.33 (95% CI 1.26–1.41), respectively.

**Table 2:** Unadjusted and adjusted hazard ratios for cause-specific survival among adult patients in the SEER database diagnosed esophageal squamous cell carcinoma, 1973 and 2013.

	Unadjusted	Model 1
	HR^a^ (95% CI^b^)	HR (95% CI)
**Race**		
White	Ref^c^	Ref
Black	1.15 (1.11–1.20)	1.08 (1.03–1.13)
Asian/Pacific Islander	0.97 (0.91–1.04)	1.00 (0.93–1.07)
**Age**		
18–49	Ref	Ref
50–59	1.08 (0.99–1.17)	1.05 (0.96–1.14)
60–69	1.06 (0.98–1.14)	1.07 (0.99–1.17)
70–79	1.10 (1.02–1.19)	1.15 (1.06–1.26)
> = 80	1.31 (1.19–1.43)	1.43 (1.30–1.59)
**Sex**		
Male	Ref	Ref
Female	0.86 (0.82–0.89)	0.87 (0.83–0.91)
**Date of Diagnosis**		
2000–2013	Ref	Ref
1990–1999	1.12 (1.07–1.18)	1.33 (1.26–1.41)
1980–1989	1.30 (1.24–1.37)	1.59 (1.51–1.68)
1973–1979	1.60 (1.52–1.69)	2.05 (1.93–2.19)
**Partnered**		
Partnered^d^	Ref	Ref
Unpartnered^e^	1.19 (1.14–1.24)	1.17 (1.12–1.22)
**Ethnicity**		
Non-Spanish/Hispanic/Latino	Ref	Ref
Spanish/Hispanic/Latino	0.92 (0.83–1.02)	0.98 (0.88–1.09)
**Staging**		
In-situ	Ref	Ref
Localized	2.99 (2.24–3.99)	3.00 (2.18–4.12)
Regional	3.76 (2.82–5.01)	4.70 (3.42–6.45)
Distant	6.81 (5.11–9.09)	6.88 (5.01–9.45)
**Radiation**		
Yes	Ref	Ref
No	1.34 (1.28–1.39)	1.78 (1.70–1.87)
**Surgery**		
Yes	Ref	Ref
No	1.81 (1.73–1.90)	2.17 (2.05–2.28)

^a^Hazard ratio;

^b^Confidence interval;

^c^Reference group;

^d^Partnered: married or unmarried/domestic partner;

^e^Unpartnered: single, separated, divorced or widowed

After stratification according to decade of diagnosis, the HR for black patients compared with white patients was 1.14 (95% CI 1.02–1.29) in 1973–1979 and 1.12 (95% CI 1.03–1.23) in 1980–1989. These disparities were not observed after 1990; the HR for black patients compared with white patients was 1.03 (95% CI 0.93–1.13) in 1990–1999 and 1.05 (95% CI 0.96–1.15) in 2000–2013. Figs 2, 3, 4 and 5 display the adjusted survival curves by race for each decade of diagnosis.

## Discussion

Overall, there appeared to be racial disparities in survival among patients with esophageal SCC patients when comparing white and black patients. However, in the stratified analyses, this disparity seemed to disappear after 1990. There was no observed disparity when comparing white and API patients.

Previous retrospective cohort studies mainly investigated the racial disparities between black and white patients [7, 9, 10–15] and occasionally, included Hispanics as well [11]. Only two studies included Asians/Pacific Islanders, one conducted between 1930 and 1967 [16] and the other one in British Columbia, Canada [17]. Many of the studies used the SEER database for their study, with years ranging from 1988–2003, 1991–2000 and 1973–1998 [7, 9, 13]. Our study is partially in agreement with previous scientific evidence that black patients with esophageal SCC have worse survival when compared to white patients. The majority of the studies showed that non-white racial and ethnic groups with esophageal cancer had lower survival rates when compared to white patients [7, 9–11]. One study found a 37% vs 60% 5-year survival rate in blacks vs whites, respectively [7]. Another revealed that age-adjusted mortality for black patients was nearly twice that of whites with a relative risk of 7.79 vs 3.96 [9]. Baquet et al included patients from the US Veterans Affairs (VA), and showed that mortality rates were increased for black patients with esophageal SCC, (Relative Risk 1.33), but not adenocarcinoma [10].

We found that survival was significantly lower for black patients only before the 1990s. This finding is somehow surprising as access to care has not improved much for blacks [19]. Inadequate health care access and insurance coverage are major factors that contributed to racial and ethnic disparities before the implementation of the affordable health care act (ACA) in 2014 [20–22]. In addition, it has been shown that even if blacks have access to care, it may not be timely and of good quality. Thus, the upcoming years will show whether the ACA will benefit racial and ethnic minorities who historically have experienced lower coverage rates and suboptimal access to care [21, 22].

As the decade of diagnosis progressed, overall survival improved, indicating a general improvement in treatment of esophageal SCC over time. Treatment and management protocols for esophageal cancer are regularly updated according to the best scientific evidence [23, 24]. The most recent, 7^th^ edition of the AJCC Cancer Staging Manual for esophagus and esophagogastric junction cancers was developed based on a database of 4,627 esophagectomy patients who were not treated with induction or adjuvant therapy [24, 25]. The treatment of early-stage esophageal cancer and high-grade dysplasia of the esophagus has changed significantly in recent years [23]. Many early tumors that were traditionally treated with esophagectomy can now be resected with endoscopic therapy alone [23]. Finally, it has to be kept in mind that, it is difficult to assess when and whether the healthcare providers in the 13 SEER registry states adopt new treatment and management guidelines for esophageal SCC patients.

The finding of a substantial decrease in survival for those with increasing stage at diagnosis was unsurprising. Moreover, the findings that radiation and surgery improve survival are consistent with previous research in this patient population [7, 8]. Thus, radiation and surgery should be offered to patients who would benefit from this treatment to increase their survival time. Many previous studies also reported poorer outcomes (besides mortality) and treatment management in racial minorities compared with whites [7, 8]. Moreover, surgical interventions such as esophagectomies were conducted less in Hispanics and blacks [7, 10, 12]. Proportions of unknown regional lymph nodes status and pathologic review were higher among blacks than whites [8]. Blacks and Hispanics were also found to have a higher incidence of esophageal cancer and diagnosed at a later stage [7, 9, 13]. Numerous factors were speculated to play a role in such disparity in mortality—such as health care access, biological behavior of cancer, variation in socioeconomic status, lifestyle differences, alcohol consumption, nitrosamines, dietary deficiencies, and environmental exposures [14].

The lower survival among unpartnered SCC patients is consistent with the current scientific evidence. Previous studies have shown that married individuals are generally healthier than unmarried individuals, which includes people who were divorced, separated, widowed, never married, or living with a partner [26–28]. The general hypothesis between these studies was that married patients have a greater social support system, which improves overall health maintenance, including medication adherence [29]. Moreover, married patients seem to have a lower mortality compared to widowed patients, possibly due to greater social support and decreased stress [30].

Limitations of our study are worth noting. First, the SEER registry only includes data from 13 US states, which are not completely representative of the US population. Asian and Pacific Islanders may be very different in regard risk factors and genetic background. However, as the definitions used by SEER for Asian/Pacific Islanders were not used consistently over time, they could not be analyzed separately. It is noteworthy to mention that most studies indeed analyze them as a combine category allowing some comparisons between the results of previous studies with ours. Additionally, we were unable to adjust for insurance status in our final model. The SEER database only included data for those patients in the database from 2007 onwards. When we analyzed this sub-sample (n = 1802), using all covariates present in our final model except decade of diagnosis and adjusting for insurance status, results comparing black and white patients did not achieve statistical significance (HR 1.02; 95% CI 0.88–1.19). Furthermore, SEER does not provide important information related to lifestyle habits such as pack-year of smoking or known social determinants of health, including variables such as socioeconomic status and education. Insurance status and other social determinants may be the underlying factors in racial disparities of esophageal SCC survival, and future research should seek to explore these factors more fully. Finally, an important limitation using SEER data is that treatment data in the SEER registry is not complete and thus, some degree of bias cannot be ruled out.

In conclusion, black patients with esophageal SCC were found to have a higher hazard of death compared to white and API patients. Survival disparities between races appear to have decreased over time. Future research that takes insurance status and other social determinants of health into account should be conducted to further explore possible disparities by race.

## Supporting information

S1 File Data access. The data underlying this study are third party data. The authors gained access to the data by submitting a request to the National Cancer Institute’s Surveillance, Epidemiology, and End Results (SEER) Program through their website: https://seer.cancer.gov/seertrack/data/request/. Interested researchers may apply for access to these data in the manner described.
(DOCX)
