# Supplementary material for: Racial disparities in cancer-related survival in patients with squamous cell carcinoma of the esophagus in the US between 1973 and 2013
Source: PLoS One. 2017 Aug 23;12(8):e0183782. doi: 10.1371/journal.pone.0183782 (PMC5568373; doi:10.1371/journal.pone.0183782)
Supplement: S1 File — The data underlying this study are third party data. The authors gained access to the data by submitting a request to the National Cancer Institute’s Surveillance, Epidemiology, and End Results (SEER) Program through their website: https://seer.cancer.gov/seertrack/data/request/. Interested researchers may apply for access to these data in the manner described. (DOCX) [file pone.0183782.s001.docx]

**1.Please confirm that the data underlying your study are third party data. PLOS defines third party data as data that are not owned by the authors.**

Confirmed

**2. Do the authors have special access privileges to these data? In your response, please describe the manner in which the authors applied for and were granted access to these data.**

The authors gained access to the data by submitting a request to the National Cancer Institute’s Surveillance, Epidemiology, and End Results (SEER) Program through their website: https://seer.cancer.gov/seertrack/data/request/

**3. Please confirm that interested researchers may apply for access to these data in the manner described.**

Confirmed.

**4. Please provide contact information for a non-author individual affiliated with the agency that owns these data to whom queries may be submitted.**

Other interested researchers may request access to the data from the SEER website: https://seer.cancer.gov/seertrack/data/request/
